# Identification of new cell size control genes in *S. cerevisiae*

**DOI:** 10.1186/1747-1028-7-24

**Published:** 2012-12-12

**Authors:** Huzefa Dungrawala, Hui Hua, Jill Wright, Lesley Abraham, Thivakorn Kasemsri, Anthony McDowell, Jessica Stilwell, Brandt L Schneider

**Affiliations:** 1 Department of Cell Biology and Biochemistry, Texas Tech University Health Sciences Center, 3601 4th St Rm. 5C119, Lubbock, TX, 79430, USA; 2 Texas Tech University, Howard Hughes Medical Institute, Lubbock, TX, USA

**Keywords:** Yeast, Cell cycle, Cell size, Growth, Cyclins

## Abstract

Cell size homeostasis is a conserved attribute in many eukaryotic species involving a tight regulation between the processes of growth and proliferation. In budding yeast *S. cerevisiae*, growth to a “critical cell size” must be achieved before a cell can progress past START and commit to cell division. Numerous studies have shown that progression past START is actively regulated by cell size control genes, many of which have implications in cell cycle control and cancer. Two initial screens identified genes that strongly modulate cell size in yeast. Since a second generation yeast gene knockout collection has been generated, we screened an additional 779 yeast knockouts containing 435 new ORFs (~7% of the yeast genome) to supplement previous cell size screens. Upon completion, 10 new strong size mutants were identified: nine in log-phase cells and one in saturation-phase cells, and 97% of the yeast genome has now been screened for cell size mutations. The majority of the logarithmic phase size mutants have functions associated with translation further implicating the central role of growth control in the cell division process. Genetic analyses suggest *ECM9* is directly associated with the START transition. Further, the small (*whi*) mutants *mrpl49Δ* and *cbs1Δ* are dependent on *CLN3* for cell size effects. In depth analyses of new size mutants may facilitate a better understanding of the processes that govern cell size homeostasis.

## Introduction

Cell size homeostasis is physiologically important to nearly all organisms. This is evident from the uniformity and conservation of size within a cell lineage amongst the individuals of a species from bacteria to man [1]. Moreover, studies in flies, mice and humans indicate the presence of an organ size checkpoint during developmental stages [2]. To ensure a population of cells that maintain a constant average cell size, it is essential that cells coordinate the processes of growth, which increases cell size, and cell division, which reduces cell size [3]. Irregularities in these processes affect fitness and function [4]. In the budding yeast *S. cerevisiae*, coordination of growth and division occurs at START, the point of commitment to the cell cycle [5,6] which is equivalent to the Restriction point in mammalian cells [7]. At START, a cell transits, essentially irreversibly, from G1- to S-phase. Early genetic analyses of START revealed that blocking cell growth prevents cell cycle progression [5]. However, the converse is not true [5]. For example, the discovery of cell division cycle (*CDC)* genes, a class of essential genes involved in cell cycle control, established that cell growth is a continuous process that proceeds unabated even when cell cycle progression is halted [8]. The end result is the production of abnormally large cells [5]. Thus, the mechanisms that regulate the cell cycle can have a profound impact on cell growth and vice versa.

Physiological studies in yeast and mammalian cells suggest that cells undergo exponential growth throughout the cell cycle [4,9-12]. Since exponential growth is inherently tied to cell size (e.g. larger cells grow faster than smaller cells), some type of “size sensing” mechanism is required for cell size homeostasis [4,13,14]. While the mechanism remains somewhat obscure, evidence suggests that in yeast commitment to division is linked to cell size [4,13,14]. In yeast, cells must attain a certain “critical cell size” before commitment across START [5,15], but while there are hints of a “size sensing” mechanism in mammalian cells, it is less clear if a similar “critical cell size” phenomenon exists in mammalian cells [9,16-20]. Nonetheless, the investigation of cell size mutants has provided mechanistic clues to START regulation in yeast. For example, some of the very first cell size mutants in yeast implicated cyclins and cyclin dependent kinases (Cdks) in the control of cell size [21-23]. These included mutants that stabilized cyclins (e.g. *CLN3-1*) in budding yeast or promoted the activity of Cdks in fission yeast (e.g. *wee1*) [22-25]. Subsequently, genome-wide genetic screens systematically identified yeast mutants with altered cell size phenotypes [26,27]. These studies led to the identification of genes which play a strong role in regulating cell cycle progression past START [26,27]. Mutants which alter *CLN* expression strongly alter cell size phenotypes. For example, deletion of the START inhibitor *WHI5* results in a population of cells with a small size phenotype [26,27]. Conversely, deletion of *CLN3* or *BCK2*, upstream activators of *CLN* expression cause a cell cycle delay thus inducing a large cell size phenotype [28,29]. Deleting both *CLN3* and *BCK2* results in inviability, but *cln3Δbck2Δ* cells can be partially rescued by inducing *CLN2* expression ectopically or by deleting *WHI5*[29-32]. Deletion of the transcription factors that regulate *CLN* transcription (e.g. *SWI4* and *SWI6)* also results in a large cell size phenotype [33]. In contrast, early *CLN* expression advances cell cycle progression and reduces cell size [31,32]. Thus, many genes involved in cell size control appear to interface with the mechanisms that regulate progression past START in budding yeast [26].

Cell size is sensitive to the conditions of external environment. Size homeostasis mechanisms exist during nutritional up shift, that are distinctly different from those involved in steady-state environment conditions [34]. Cells cultured in poor nutrients grow slower and are smaller compared to isogenic populations cultured in rich environmental conditions [35-38]. As such, ribosome biogenesis has been strongly implicated in modulating critical cell size for yeast cells at START [37,39,40]. Furthermore, genes implicated in the process of ribosome biogenesis are also size mutants [26,27]. Indeed, a recent report has established multiple genes that function in protein synthesis as strong regulators of START [39]. Interestingly, the majority of mutants that altered cell cycle progression did not affect cell size and vice versa [39]. Despite these observations, evidence suggests that carbon source modulates size via Clns [41,42], and that growth rates are potentially linked to *CLN* thresholds for START entry [38,43,44]. These results warrant further investigation into the mechanistic regulation of cell division by genes affecting growth and cell size which would help elucidate the relationship between nutrient transduction signals and cell cycle entry. Also, nutrient sensing pathways play an important role in modulating the aging process in various model systems [45]. Thus, it would be beneficial in elucidating the coordination between growth and proliferation under different nutritional environments.

The basic mechanisms of cell cycle control are well conserved evolutionarily. Not only is gene function highly conserved, but the products of these genes also appear to have the same fundamental role in the regulation of cell size from yeast to man [1]. Indeed, an analogous system for G1-S transition exists between yeast and mammals wherein Cln3, SBF and Whi5 play similar roles to that of cyclin D, E2F and RB respectively [46-48]. Moreover, like their yeast homologs, the expression of cyclin D, E2F and RB influences cell size homeostasis. For example, cells lacking cyclin D are larger than normal while cells over-expressing cyclin D are smaller than normal [49-53]. Moreover, like *whi5Δ* strains, cells lacking pRb are smaller than normal [54-56]. Conversely, loss of E2F function increases cell size [57]. The extent of evolutionary conservation of cell cycle genes between yeast and mammals signifies the importance of cell size control studies in *S. cerevisiae*. Although the genetic pathways involved in cell cycle control are well established, the mechanisms whereby these same pathways modulate cell size are not well understood. Therefore, the elucidation of gene function in yeast is likely to provide valuable insights into mammalian cell biology.

For this study, we screened the entire yeast knock-out collection version 2 (YKOv2) containing 779 ORF deletions for cell size mutants. From this screen, 10 new strong size mutants were identified: nine from logarithmic and one from saturation cultures. Like previous screens, the majority of the size mutants are involved in some aspect of the translation process. This further implicates the control of translation in the mechanisms that coordinate growth and proliferation, and completion of this screen will provide a valuable database for researchers interested in dissecting the process of cell size control.

## Results

### Cell size screen analysis

In the two previous studies, 5958 diploid deletion strains were screened for cell size mutants in saturated cultures [27] while 4812 haploid deletion strains were analyzed in log-phase cultures [26]. In total, ~90% of the 6607 currently annotated yeast ORFs were evaluated for cell size defects [26,27,58]. To expand upon these results, the second generation yeast gene deletion strain collection (YKOv2) was obtained from OPEN Biosystems. These included all the additions/updates (e.g. new ORF annotations that were added to the existing database) and corrections to the previous collection [59,60]. Thus, a total of 779 diploid strains were sized in both logarithmic and saturated cultures to identify new cell size mutants (Additional file 1: Table S1). Amongst these strains were 435 new ORF deletions (235 essentials and 200 non-essentials constituting ~7% of the genome) which have not been sized previously (Additional file 1: Table S1). Consequently, after this current screen, ~97% of the available yeast ORFs have now been screened for cell size mutants.

**Figure 1:**
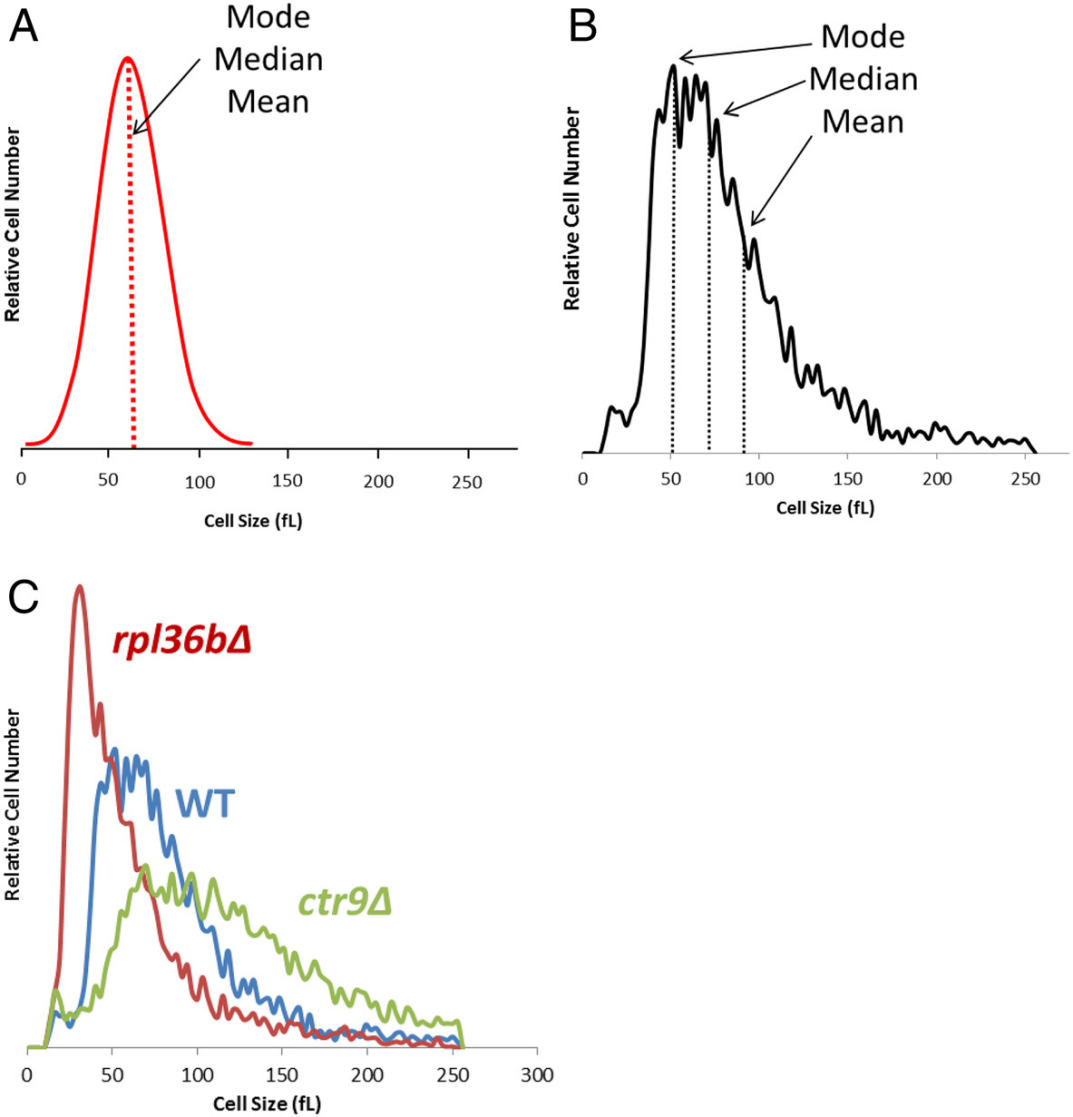
**Size distribution curves for budding yeasts. (A)** Curve depicts a normal distribution curve with similar mean, median and mode values. **(B)** Curve depicts a size distribution curve for budding yeasts obtained in logarithmic phase. Unlike normal distributions, yeast cell size curves are usually skewed to the right where the mode is often the smallest value, since ~50% of the population is comprised of newly born daughter cells. Also, due to the asymmetric nature of cell division and exponential nature of cell growth, the mean value tends to be larger than the median. **(C)** Coulter counter curves for representative size mutants in the logarithmic phase: *whi* mutant (*rpl36bΔ*), wild-type (WT) and *uge* mutant (*ctr9Δ*).

The size curves of budding yeast cultures are usually not normal distributions (Figure 1A), but rather are positively skewed to the right (Figure 1B). Therefore, we collected and compared average mean, median and mode size values as previously described [27]. To ensure the proper identification of the size mutants, outliers were selected as potential size mutants on the basis of being +/− 2 standard deviations from average mean, median and mode values (see Materials and Methods). This methodology was applied to both logarithmic and saturation phase readings (Additional file 2: Figure S1). Evaluation of mean cell sizes from the entire population of logarithmic and saturated cultures revealed a distribution curve that was nearly identical to our previous study (Additional file 3: Figure S2 and data not shown). In addition, screenings identified 10 new deletion strains as potential size mutants: 9 in logarithmic phase and 1 in saturation phase. In total in log phase, seven gene deletions (*rpl36bΔ, mrpl49Δ, cbs1Δ, rpl42aΔ, rom2Δ, rpl16bΔ,* and *yjr114wΔ*) produced abnormally small (*whi*) cells while two gene deletions (*ctr9Δ* and *ecm9Δ*) increased cell size (*uge* mutants) in log phase (Table 1). Only one gene deletion, *ctr9Δ*, was found to significantly alter size in saturation and produced abnormally large cells (Table 1). Representative cell size plots for a *whi* mutant, *rpl36bΔ* and an *uge* mutant, *ctr9Δ* are shown in Figure 1C.

**Table 1:** Summary of cell size mutants

	**Gene**	**ORF**	**Cell size (fL)**	**Budding %**	**G1%**	**S/G2/M%**	**Human homolog**	**E-value**	**Function**
***LOGARITHMIC***									
	WT		101.7	56	38	62			
***whi***	*rpl36bΔ*	YPL249C-A	66.0**	40	62	38	RPL36	2e-26	Translation
	*mrpl49Δ*	YJL096W	66.8**	45	59	41	RFT1	0.16	Translation
	*cbs1Δ*	YDL069C	71.2**	43	45	55	TLR5	0.28	Translation
	*rpl42aΔ*	YNL162W	73.9**	46	50	50	RPL36AL	2e-51	Translation
		YJR114W	77.1*	54	53	47	-	--	Unknown
	*rom2Δ*	YLR371W	80.2**	60	55	45	ARHGEF3	3e-16	GEP for Rho
	*rpl16bΔ*^#^	YNL069C	83.5*	52	44	56	L13a	5e-76	Translation
***uge***	*ecm9Δ*	YKR004C-A	135.9*	62	37	63	KCNS2	0.060	Cell Wall Organization
	*ctr9Δ*	YOL145C	128.5*	36	33	67	CTR9	2e-48	Transcription
***SATURATED***									
	WT		49.7	3	96	4			
***uge***	*ctr9Δ*	YOL145C	76.8*	11	93	7	CTR9	2e-48	Transcription

A summary of cell size mutants, grouped together under logarithmic and saturated conditions, is shown. Data includes the gene, open reading frame (ORF) of the gene, mean cell size in femtoliters (fL), budding index values, cell cycle distribution in logarithmic phase obtained by FACS, human homolog, E-values and cellular function associated with each gene. Statistical differences in mean cell size values were calculated using unpaired *t*-test (**p<0.0001, *p<0.05). ^#^ All genetic strains are homozygous diploids except for *rpl16bΔ* which is a heterozygous diploid. Thus, *rpl16bΔ* is a haploinsufficient cell size mutant. Note that YJR114W is a dubious ORF.

Internal controls within the second generation deletion collection allowed us to test the reproducibility of previous cell size measurements. For example, of the 779 deletion strains in YKOv2, 144 were newly constructed deletions of previously knocked-out genes [60]. Importantly, sizing of these new deletions led to the identification of four new size mutants (*cbs1*Δ*, rpl16b*Δ*, rom2*Δ*, rpl42a*Δ) suggesting that the original deletions might not have completely removed the function from these ORFs. However, 132/140 (94.2%) of the remaining re-made deletions (internal controls) reproduced the expected size phenotype (Additional file 1: Table S1). These included 10 previously reported cell size mutants [26,27] (Additional file 1: Table S1). Of the eight deletions that failed to reproduce the expected size phenotype, two were very close to being significantly larger (Additional file 1: Table S1). Thus, the true reproducibility likely ranges from 94-96% indicative of the robustness of the approach.

### Mutants alter daughter birth size and “critical cell size”

**Figure 2:**
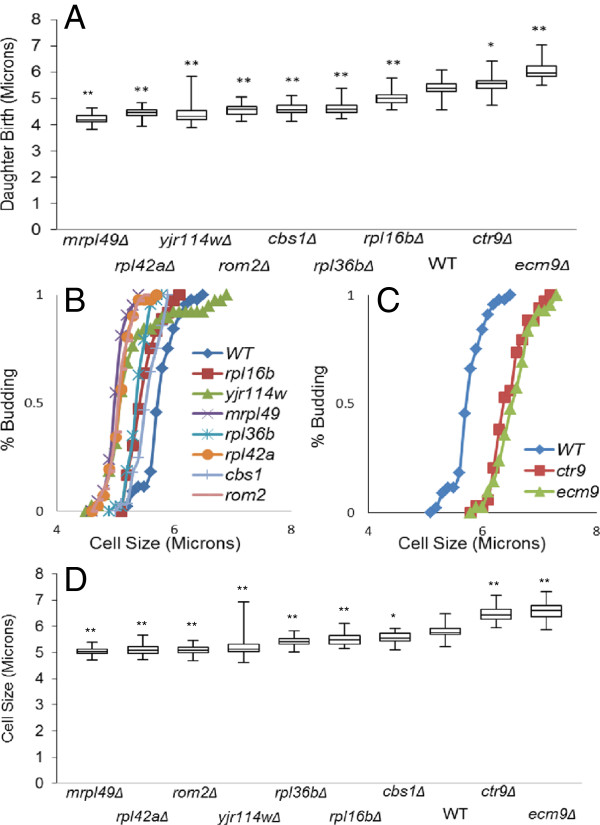
**Mutants alter birth size and “critical cell size” for daughters.** (**A**) Box plot (refer Materials and Methods) represents the cell size distributions (in microns) at which daughter cells were born. Average size: *whi* mutants *mrpl49Δ*=4.2, *rpl42aΔ*=4.5, *yjr114wΔ*=4.5, *rom2Δ*=4.6, *cbs1Δ*=4.6, *rpl36bΔ*=4.6, *rpl16bΔ*=5, WT=5.4 and *uge* mutants *ctr9Δ*=5.5, *ecm9Δ*=6.1 (**p<0.0001, *p<0.05). **(B)** Plot represents % budded virgin daughters vs. cell size (microns): the *whi* mutants undergo division at an average “critical cell size” (defined by 50% budded) that is smaller than that of the wild type (*mrpl49Δ*= 5.0, *rpl42aΔ*= 5.1, *rom2Δ*= 5.1, *yjr114wΔ*=5.3, *rpl36bΔ*= 5.4, *rpl16bΔ*=5.5, *cbs1Δ*= 5.6, WT= 5.8). **(C)** Plot represents % budded virgin daughters vs. cell size (microns): large cell (*uge*) mutants undergo division at an average “critical cell size” larger than that of the wild type (WT= 5.8, *ctr9Δ*= 6.5, *ecm9Δ*= 6.6). **(D)** Box plot represents distributions (n=30-40) of daughter cell sizes at which they bud (**p<0.0001, * p=0.0008). Statistical differences were determined by Mann Whitney Test with p=0.05 as cutoff value.

Analyses of Coulter Counter data only provides size data for the entire population. To evaluate how the size of individual cells vary, we used time lapse photography of single cells over 10–12 hours as previously described [61]. Examination and sizing of individual cells revealed that all seven *whi* mutants produced virgin daughter cells that are statistically smaller (p<0.0001) than wild type virgin daughter cells (Figure 2A). In addition, deletion of *CTR9* and *ECM9* produced statistically larger than normal virgin daughter cells (p<0.05) (Figure 2A).

**Figure 3:**
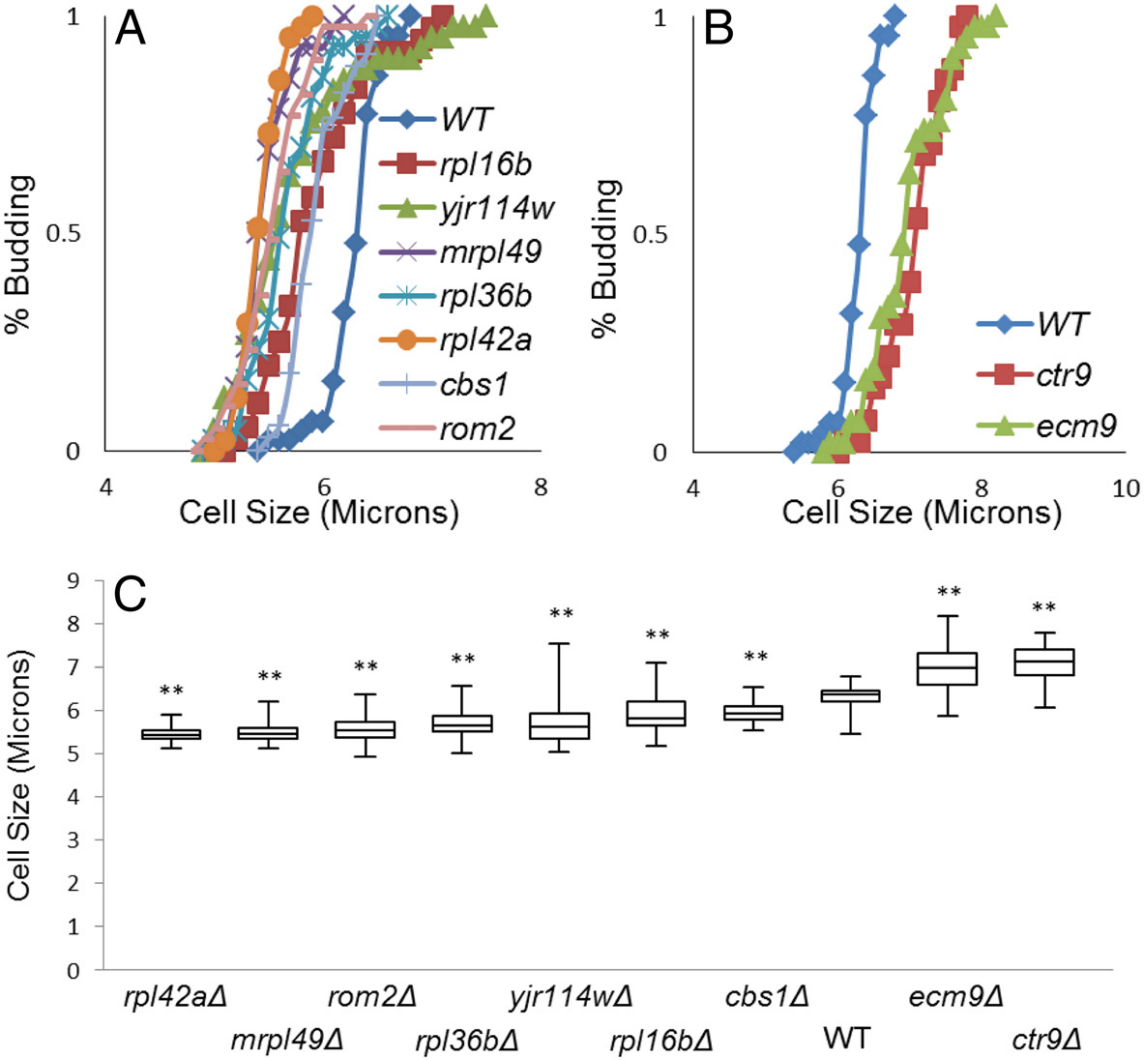
**Mutants alter “critical cell size” at START for mothers.(A)** The % of budded mother cells vs. cell size (microns) is plotted: *whi* mutants undergo division at a “critical cell size” smaller than that of the wild type (*rpl42aΔ*= 5.5, *mrpl49Δ*= 5.5, *rom2Δ*= 5.6, *rpl36bΔ=* 5.7, *yjr114wΔ*= 5.7, *rpl16bΔ*= 5.9, *cbs1Δ*= 6.0, WT= 6.3). **(B)** The % of budded mother cells vs. cell size (microns) is plotted: large cell (*uge*) mutants undergo division at a “critical cell size” larger than that of the wild type (WT= 6.3, *ecm9Δ*= 7.0, *ctr9Δ*= 7.1). **(C)** Box plots represent size distributions (n=35-45) of mother cell populations at which they bud (**p<0.0001). Statistical differences were determined by Mann Whitney Test with p=0.05 as cutoff value.

In yeast, cells commit to division after attaining a certain “critical cell size”. To observe changes in the “critical cell size” at START, time-lapse microscopy was also used to study the pattern of cell division over time for the new size mutants. For experimental purposes, size at bud emergence was measured for daughter and mother cells and plotted against % budded. START usually refers to the point at which 50% of the cell population has budded. For all the seven *whi* mutants, the size of virgin daughters at START was significantly smaller (p<0.0001) than in wild type cells (Figure 2B and 2D). In addition, for all seven *whi* mutants, mother cells progressed past START at a significantly smaller size compared to wild type mother cells (Figure 3A and C). For the large cell mutants, the situation was exactly the reverse; virgin daughters were born large (Figure 2A), and both daughters (Figure 2C and 2D) and mothers (Figure 3B and C) progressed past START at a cell size that was significantly larger (p<0.05) than in wild type cells. These results suggest that the newly identified cell size mutants alter the “critical cell size” at which commitment to cell division occurs.

### Cell cycle effects

Many of the known cell size control genes also strongly affect cell cycle progression [22,29,31,32,62,63]. Thus, flow cytometry and the budding index of cultures were used to assess cell cycle distributions in the newly identified cell size mutants (Table 1). In addition, we were able to directly measure the length of the unbudded (G1-phase) and budded (S-G2-M-phases) segments of the cell cycle in all of the cell size mutants from the time lapse studies of single cells (Table 2). From these data, several trends emerged. First, overall cell cycle time was increased in all mutants regardless of their size (Table 2). Second, a considerable increase in the length of G1-phase was predominantly responsible for the increase in cell cycle times (Table 2). Third, while G1-phase was increased in all mutants, the degree to which it was affected was extremely variable: ranging from a 0–7.6 fold increase in mother cells or a 1.3-5.4 fold increase in daughters (Table 2). Appropriately, mutants with the shortest G1-phases had, in general, the highest budding indices (Tables 1 and 2). In this respect, only one *whi* mutant, *rom2Δ*, increased the budding percentage compared to that of wild type which would suggest a putative inhibitory role of *ROM2* in the START transition. In contrast, the majority of the *whi* mutants had an increased accumulation of cells in G1-phase compared to the wild type. Such a pattern is characteristic of cells with a slow growth phenotype [35] in which cells display a small size phenotype with extended periods of G1 phase [64].

**Table 2:** Summary of cell cycle effects

	**Daughter**					**Mother**						**Overall**	
**Strain**	**Birth size**	**B1 size**	**G1 (min)**	**B2 size**	**CT**	**B1 size**	**CT1**	**B2 size**	**CT2**	**B3 size**	**G1 (min)**	**CT**	**RGR**
WT	5.4	5.8	32	6.0	83	6.3	75	6.4	74	6.4	4	77	100%
*mrpl49Δ*	4.2	5.1	172	5.2	167	5.5	140	5.6	154	5.7	30	154	51%
*rpl42aΔ*	4.4	5.1	94	5.4	131	5.5	97	5.5	115	5.6	8	114	58%
*yjr114wΔ*	4.5	5.4	136	5.5	183	5.8	136	5.9	137	6.0	25	152	67%
*rpl36bΔ*	4.6	5.4	131	5.6	158	5.7	143	5.8	139	5.8	24	147	67%
*rom2Δ*	4.6	5.1	89	5.3	135	5.6	115	5.6	111	5.7	4	120	64%
*cbs1Δ*	4.6	5.6	141	5.9	208	6.0	129	6.1	150	6.2	26	162	69%
*rpl16bΔ*	5.0	5.5	47	5.8	124	5.9	94	6.0	100	6.1	6	106	83%
*ctr9Δ*	5.5	6.5	130	6.8	153	7.1	110	7.2	145	7.3	20	136	116%
*ecm9Δ*	6.1	6.6	41	7.0	127	7.0	114	7.2	107	7.3	8	116	155%

Data gathered from microscopic analyses of >30 individual cells over a 6–10 hour time course is presented. Cell diameters are in microns. In each case, the birth diameter of daughter cells is shown. In addition, the size at which cells bud for the first time (B1) and subsequent buds (B2, etc.) are also provided. The length of G1-phase is directly measured as the time cells remain unbudded. Cycle time (CT) is measured from the time a cell first buds until its subsequent bud. Overall cycle time (CT) is shown as the average of one daughter CT and the first two mother cell cycle times. The relative growth rate (RGR) is a measure of the total cell volume produced per generation and is determined by calculating the change in volume of mother cells in each generation added, to the volume of daughter cells produced. For volume calculations, cells are assumed to resemble spheres and the formula 4/3πr^3^ is used. Wild type (WT) cells are set to 100% and all mutants are subsequently normalized to this value.

From the *uge* mutants identified, *ctr9Δ* strongly reduces the budding index values to that of the wild type while *ecm9Δ* moderately increased budding in log phase (Table 1). In contrast, the *uge* mutant *ctr9Δ* in saturated phase increased the percentage of budded cells and concomitantly decreased the number of cells in G1-phase (Table 1) suggesting that these mutants impeded the ability of cells to exit the cell cycle.

In order to establish any potential hierarchical role of the newly identified cell size mutants in START regulation, double deletion combinations for individual mutants were created with the START activator *CLN3*; and the START inhibitor, *WHI5*. In wild type cells, deletion of *CLN3* results in a cell cycle delay thereby inducing a large cell size phenotype [26,27]. We found that deletion of *YJR114W*, *RPL36B*, *ROM2* and *RPL42A* yielded an intermediate size phenotype in combination with deletion of *CLN3* (Table 3). However, deletion of *MRPL49* and *CBS1* did not reduce the size of *cln3Δ* cells. These results suggest that the small size phenotype of the *mrpl49Δ* and *cbs1Δ whi* mutants is dependent upon *CLN3.* With respect to the *uge* mutants, both *ctr9Δcln3Δ* and *ecm9Δcln3Δ* double mutants were larger than either haploid alone indicating a synergistic effect (Table 3 and Additional file 1: Table S1). On the other hand, combination of *whi5Δ* with all the *whi* mutants (except *rpl42aΔ*) resulted in double mutants that were smaller than either haploid alone indicative of a synergistic effect. With respect to the *uge* mutants, *ctr9Δwhi5Δ* double mutant displayed an intermediate size phenotype while *ecm9Δwhi5Δ* double mutant was small. Since *whi5Δ* was epistatic to *ecm9Δ*, the large size phenotype of *ecm9Δ* mutant is most likely dependent upon *WHI5* (Table 3 and Additional file 1: Table S1).

**Table 3:** Genetic epistasis analyses

	***cln3Δ***	***whi5Δ***
***mrpl49Δ***	↑	●
***yjr114wΔ***	+	●
***rpl36bΔ***	+	●
***rpl42aΔ***	+	**-**
***cbs1Δ***	↑	●
***rom2Δ***	+	●
***rpl16bΔ***	NA	NA
***ecm9Δ***	●	↑
***ctr9Δ***	●	+

Genetic epistasis analysis with *cln3Δ* and *whi5Δ*. Double deletion combinations were created for the new logarithmic phase size mutants with *cln3Δ* (*uge* mutant) and *whi5Δ* (*whi* mutant). Haploids were analyzed for the cell size phenotype. Table represents the pair wise genetic relationships: Intermediate (+), epistatic (↑) (the gene pointed to by the arrow masked the size phenotype of the other deletion), synergistic (•), no effect (−). Epistasis was operationally defined as an average cell size for double mutants within +/− 5% of size of either single mutant. Synergistic effects refer to combinatorial cell sizes that are either substantially larger or smaller than each single mutant. Intermediate refers to combinatorial cell sizes that lie between the size of each single mutant. Since *RPL16B* is an essential gene, it could not be analyzed in these studies. Size values with statistics can be found in Additional file 1: Table S1.

Finally, over-expression studies were carried out to determine whether any of the newly identified cell size mutants could function as activators or inhibitors of START. Over-expression of *ECM9* resulted in a strong reduction of cell size distribution (Table 4). Furthermore, the increased budding index values along with a higher percentage in S/G2/M phase suggests that Ecm9 promotes START (Table 4). On the other hand, over-expression of *CTR9* increased the budding index values without a concomitant decrease in cell size of the culture. Counter-intuitively, over-expression of six *whi* mutants also reduced cell size (Table 4). Evidence indicated that over-expression of these *whi* mutants led to decreased proliferation rates in the majority of cases (data not shown) and some G1-phase delays (Table 4).

**Table 4:** Over-expression analyses

**WT +**	**Mean**	**N**	**SE**	**G1%**	**S/G2/M%**	**Budding index**
**Empty Vector**	78	11	1	56.99	40.56	44.3
***ECM9***	62**	11	1	52.21	47.79	60.3
***CTR9***	80	14	1	54	46	65.7
***MRPL49***	68**	11	2	52.38	47.62	51.9
***YJR114W***	68*	13	2	72.53	27.47	44.4
***RPL42A***	68**	13	1	52.24	47.76	57.7
***RPL16B***	72**	13	1	44.31	55.69	62.3
***CBS1***	69*	14	2	72.64	24.46	45.7
***RPL36B***	73	6	4	59.31	40.09	41.6

Over-expression analysis in wild type cells. This table illustrates the effects of over-expressing the new size mutant genes in wild type yeast cells. Data includes the average cell size from logarithmic cultures, number of replicates, standard error, % of cells in G1 and S/G2/M phases as determined by flow cytometry, and the budding index values. Statistical differences in mean cell size values were calculated using unpaired *t*-test (**p<0.0001, *p<0.0051). All analyses were carried out in -Ura Raf/Gal media using *GAL*-inducible vectors. Vector alone (pB63) was used as a control. Note: We were unable to generate a *GAL-ROM2* inducible vector.

### Cell growth and cell size homeostasis

Examination of the known function of the new cell size mutants suggests that reduced protein synthesis and overall growth rate may correlate with decreased cell size. For example, 5 of the 7 *whi* mutants isolated are involved in general protein translation (Table 1). Three of these mutants, *rpl36bΔ, rpl42aΔ* and *rpl16bΔ,* associate with the large ribosomal subunit in the ribosome assembly process [65] while *MRPL49* and *CBS1* are involved in mitochondrial protein synthesis [66]. Since previous screens for cell size mutants have revealed that a significant number of *whi* mutants whose gene products normally function in protein synthesis or ribosome biogenesis [26,27], the growth rate of individual cells was determined from time course data. By measuring the rate of cell size increase in mother cells and the size of buds produced, we were able to determine the average relative growth rate for both *whi* and *uge* mutants in each generation. In doing so, we found all seven *whi* mutants had average growth rates that were 17-49% less than wild type cells (Table 2). In contrast, the average growth rate was higher in both large cell mutants (Table 2). These results suggest that growth rates correlate well with cell size phenotypes.

## Discussion

### Identification and impact of new cell size mutants

Cell size homeostasis is attained by a highly intricate relationship between growth and proliferation. Previous studies suggest that growth is rate-limiting for commitment to cell division such that cells must attain a “critical cell size” prior to START transition [5,6,15]. To better understand the global mechanism behind cell size control, two systematic genome-wide genetic screens were carried out ten years ago to detect cell size mutants from both log phase and saturated cultures [26,27]. Many of the identified cell size genes are directly associated with START and are linked to *CLN*s [27]. Significantly, the yeast ortholog of the pRB tumor suppressor was identified in the previous screens [26,27]. In addition, key regulators of cell growth and ribosome biogenesis (e.g. Sfp1 and Sch9) were shown to be strong *whi* mutants [26]. Such analyses proved to be highly valuable in the detailed characterization of cell size machinery. Since the original screens were conducted, a second generation yeast deletion collection has been constructed containing hundreds of new knockout strains and re-created deletions (n.b. most of these deletions were remade to correct quality control issues such as a partial rather than complete ORF deletion). The objective of this study was to expand the genome-wide systematic screen concept by evaluating the ~800 newly made ORF deletions for size mutants. In so doing, 9 cell size mutants were identified in the logarithmic phase cultures and 1 cell size mutant was identified in saturated cultures. Of the ~140 ORF deletions that were re-made, >94% of the strains reproduced the original phenotype including both strong *whi* (e.g. *sch9Δ*) and large cell phenotypes (e.g. *ccr4Δ*). Importantly, screening of the re-made ORF deletions yielded four new size mutants, supporting the notion that some deletions in the original collection did in fact retain some gene function. In total, seven new *whi* and two new large cell mutants were identified, and after completion of this work, ~97% of the yeast genome has been now screened for cell size mutants.

In the previous screens, many of the *whi* mutants identified involved the deletion of genes that function in ribosome biogenesis and translation [26,27]. Of note, 5 of the 7 newly identified *whi* mutants are involved in the general process of translation supporting the hypothesis that robust ribosomal biogenesis is integral for cell growth and is a prerequisite for normal cell cycle progression [39,40]. One *whi* mutant, *yjr114wΔ*, has an unknown function; however its phenotype may be due to loss of function of the overlapping *RSM7* ORF. The *RSM7* gene encodes a mitochondrial ribosomal subunit that when deleted also yields a small cell phenotype [26]. A final *whi* mutant, *rom2Δ*, is also clearly involved in the regulation of cell growth. Rom2 is a GEF protein which interacts with the TOR signaling pathway in sensing nutrients from the external environment [67]. TOR is implicated to play a major role in the overall synthesis of proteins and inhibiting its activity strongly affects cell size phenotypes [68,69]. Also, Rom2 activates Rho1 GTPase and *rho1*^*ts*^ mutants have been reported to reduce the cell volume of both mother cells at G1/S transition and daughter cells at birth [70]. Interestingly, premature *CLN2* expression had been observed in *rho1-3* mutants and may help explain why *rom2Δ* mutants display a small size with higher budding index. Amongst the *uge* mutants isolated, *CTR9* plays a direct role in the transcription of *CLN*s through association with the Paf1p complex [71] and *ecm9Δ* strains exhibit alterations in cell surface biosynthesis due to defects in β 1,3-glucan synthesis [72]. Significantly, 8 of the 9 newly identified log phase mutants have putative human homologs (Table 1) suggesting that cell size control may be evolutionary conserved.

Initial investigations of the newly identified cell size mutants reveal that all of the genes identified impact cell size by altering cell growth rates. Herein, we report that critical cell size at START is reduced in 7/7 *whi* mutants in both daughter and mother cells while cell growth is also decreased in all *whi* mutants. Similarly, critical cell size at START is increased in both large cell mutants while the rate of cell growth is clearly elevated in both *ecm9Δ* and *ctr9Δ* cells. Indeed, elegant work done by Jorgensen *et al.* elucidated a potential link between ribosome biogenesis, cell growth, and START which may be predominantly responsible for the observed cell size defects [37], and a potential connection between newly identified size mutants and this work is discussed below.

A comparison between current results and the outcome of the previous two systematic cell size screens reveals striking similarities. First, as previously observed, considerably more size mutants were identified from log phase as compared to saturated cultures [26,27]. Second, most size mutants identified in log phase were not also detected as size mutants in saturated cultures [26,27]. The reason for these observations is not clear at this time but does indicate that different mechanisms impact cell size in log phase as compared to saturated cultures. Finally, the frequency of size mutants detected was very similar and with 97% of the genome screened, it is clear that ~6% of the yeast genome is involved in log phase cell size control as opposed to largely different subset of genes (only ~1% of the genome) that modulates cell size in saturation.

### At the crossroads: cell size regulation and cell cycle progression

One of the primary goals of systematic genetic size screens was to identify genes that regulate START. For example, in budding yeast, START is dependent on the activity of the G1-phase cyclin *CLN3* and its cyclin-dependent kinase Cdc28 [28,73-75]. *CLN3* can regulate the rate of accumulation of *CLN1/CLN2* and alter the “critical cell size” at START [22,23,76]. Deletion of *CLN3* results in delayed accumulation of *CLN1/CLN2* causing the cells to enter cell division at a much larger cell volume. G1-phase cyclins *CLN1/CLN2* dramatically alter the “critical cell size” and genes which regulate their transcription have been isolated [22,29,31,32,62,77,78]. Since the role of *CLN3* and *WHI5* in cell cycle commitment has been well characterized in yeast in addition to the function of their orthologs in higher eukaryotes [47], we wanted to examine the role of newly identified size mutants with START regulation. To accomplish this, we created double mutants between all newly isolated size mutants with either the deletion of an inhibitor (e.g. *whi5Δ*) or an activator (e.g. *cln3Δ*) of START. In so doing, we found that all of the *whi* mutants, except *rpl42aΔ,* had a synergistic effect on cell size when combined with *whi5*Δ (i.e. double mutants were smaller than single mutants). Most likely, the size effects in these *whi* mutants are mediated through a pathway which is independent of *WHI5.* This result infers the existence of an additional inhibitor to START. A parallel inhibitor to *WHI5* has been isolated [47], but its relationship with the newly identified *whi* mutants is not known. These results are intriguing in light that the yeast counterparts of the mammalian p16 and p21 cyclin dependent inhibitors have not yet been identified. Most of the new *whi* mutants when combined with *cln3Δ* produced intermediate size phenotypes; however, the large cell phenotype of *cln3Δ* was epistatic to *mrpl49Δ* and *cbs1Δ* suggesting that these genes function upstream of Cln3.

The large cell size of *ctr9*Δ mutant is partially dependent on the activity of *WHI5* since double mutants display additive effects. While *CTR9* (Cln Three Requiring) was first identified in a screen for mutants which required a functioning copy of *CLN3* for viability [29], unlike previous studies [71], we find that *cln3*Δ*ctr9*Δ mutants are viable albeit slowly dividing very large cells. This result is likely due to different strain backgrounds. On the other hand, over-expressing *CTR9* did not cause cell size changes but increased the budding index of the population. G1-phase cyclins regulate bud emergence in budding yeasts and localization of *CLN2* in the cytoplasm is responsible for this process [79,80]. Although *CTR9* is proposed to have a direct role in *CLN2* transcription, it is somewhat surprising that the budding index of cells increases but cell size does not decrease. However, deletion of *WHI5* in *ecm9Δ* mutants results in very small cells. These results suggest that Ecm9 functions upstream of Whi5 and may regulate START by modulating Whi5 activity. Like *cln3*Δ*ctr9*Δ mutants, *cln3*Δ*ecm9*Δ cells are also slowly dividing very large cells indicating a general delay in progression past START in *ecm9Δ* mutants. Indeed, over-expression of *ECM9* resulted in a dramatic reduction in cell size as well as a strong decrease in the percent of unbudded G1-phase cells supporting the notion that *ECM9* directly promotes progression past START.

Over-expression of Clns prematurely promotes cell cycle progression. The end result is the production of a population of small cells with a smaller percentage of cells in G1-phase. Since most cell size mutants appear to interact directly or indirectly with the START machinery (e.g. *CLN* transcription is induced at a smaller than normal size in *whi* mutants and vice versa), the logical assumption is that most *whi* mutants would advance cell cycle progression and thereby reduce the percentage of cells in G1-phase. Conversely, large cell mutants might be expected to delay cell cycle progression and thereby increase the percent of G1-phase cells. This concept was recently investigated on a genomic-wide scale. Strikingly, Hoose *et al.* found virtually no correlation between cell size mutants and cell cycle distributions [39]. For example, the majority of cells showing a dramatically increased or decreased percent of G1-phase cells were not cell size mutants [39]. Moreover, the majority of cell size mutants failed to display altered cell cycle distributions [39]. Our current results largely corroborate these findings. The apparent disconnect between cell size regulation and cell cycle progression was reinforced by our over-expression studies. While over-expression of 7/8 of our cell size mutants reduced cell size, over-expression of only 2/7 of these genes dramatically altered cell cycle distributions, which stands in contrast to another study where changes in cell cycle progression were shown to be predominantly due to gain of function alterations [81].Thus, despite the fact that most *whi* mutants appear to advance the timing of *CLN* transcription, they do not appear to advance START. The reasons and mechanisms behind this disconnect warrants considerably more investigation.

### Future perspectives: the slow growth conundrum and a role for cell size in lifespan regulation?

Evidence suggests that a minimal threshold level of Clns links cell size to START [38]. In this respect, decreasing or delaying Cln expression concomitantly slows cell cycle progression to produce abnormally large cells. Therefore, precise coordination between growth and proliferation is indispensable for cell size homeostasis. Since Clns are inherently and constitutively highly unstable proteins [82,83], steady state Cln levels would seem to be an excellent measure of the synthetic capacity of a cell and thereby provide a means to link cell growth to cell division [4,13,14,44]. As expected, Cln levels are low in slowly growing cells. However, counter-intuitively, slowly growing cells require considerably lower levels of Cln to progress past START [38]. Conversely, rapidly growing cells require high levels of Cln to bud [38]. The reasons for these observations are still not known, but a thorough investigation of the relationship between cell size mutants and the regulation of Cln expression and abundance may provide some clarity to this conundrum. Another possibility is that the rate of protein synthesis is the major determinant of cell size. Dissection of the relationship between cell size mutants, Cln expression, and cell cycle progression will be a key step in the elucidation of this issue.

Finally, recent studies have demonstrated that cell size may be relevant to the rate at which yeast cells age [61]. For example, many of the size mutants exhibit lifespan phenotypes dependent on the size at birth, i.e. small cells have an extended lifespan compared to cells that are big in size [61]. A similar correlation was obtained with size mutants obtained from this screen. *rpl42a*Δ, a *whi* mutant, had an extended lifespan compared to the wild type [61]. Moreover, two other *whi* mutants, *rom2*Δ and *rpl16b*Δ, identified in this screen are also reported to have a prolonged lifespan [84,85]. Although not all mutants that affect cell size have a lifespan phenotype, the identification of new size mutants will aid in the continued investigation into the relationship between size and replicative lifespan.

## Conclusions

Proper coordination between cell growth and proliferation is essential for normal propagation, development and differentiation. Multiple studies have outlined the significance of such coordination in cell size homeostasis. To understand the mechanisms of cell size control, two genome wide screens had been carried out to identify cell size mutants. Many genes from these screens have now been established in START regulation. To complete the initial screen and identify previously unknown cell size genes, nearly 800 new diploid strains were sized in logarithmic and saturation phase. Ten new strong cell size mutants were thus identified. Nearly all of the new *whi* mutants function in the translation process thus further supporting the integral role of growth in cell cycle commitment. Genetic analyses suggest that *CLN3* is required to mediate the size effects in *mrpl49Δ* and *cbs1*Δ small mutants. Finally, *ECM9* was found to be strongly associated with START. After completion of this study, ~97% of the yeast genome has now been screened for cell size mutants. The consistency of the type and function of the size mutants identified here reaffirms the robustness of genome wide screen approaches and augments the current and valuable database of cell size control genes.

## Materials and methods

### Cell size analysis

YKOv2, containing the homozygous and heterozygous diploid *S. cerevisiae* deletion strain sets, were obtained from OPEN BIOSYSTEMS. To assay cell size in saturation, five μl of each strain was spotted onto 96 grid points on 2% YPD plates and incubated at 30°C for 3 days. A small amount of each colony was suspended in 500μl of sterile water. Subsequently, ten μl of this dilution was re-suspended in 10ml of Isoton II (Beckman-Coulter), and cell size was determined using the Z2 Coulter Counter Channelyzer (Beckman-Coulter). For logarithmic phase cell size readings, YPD cultures containing 1–3 × 10^6^ cells/ml were grown to a density of 1-4 × 10^7^ cells/ml, and cell size was measured as discussed above. The geometric mean, median and mode values were recorded for 767 strains in the logarithmic phase and 772 strains in the saturation phase (Additional file 1: Table S1). For statistical analysis, outliers (+/− 2 standard deviations for all 3 parameters; mean, median and mode) were identified as cell size mutants. To ensure stringency, this selection was applied to data obtained from homozygous, heterozygous, and combined data (homozygous + heterozygous) for all the deletion strains (Additional file 1: Table S1). Using this approach, 32 deletion strains were initially identified as outliers. Of these, 10 strains had already been mentioned as size mutants, namely *mrpl36Δ, mrc1Δ, bub3Δ, sch9Δ, ydr417cΔ, ccr4Δ, bcm2Δ, pop2Δ, ydr433wΔ* and *bud22Δ*. From the remaining strains, ten significantly reproduced their size phenotypes after at least three independent measurements (Additional file 1: Table S1). PCR amplification of the unique barcodes was carried out to confirm the absence of the genes in the newly identified size mutants (data not shown).

### Cell cycle analysis

Cells from logarithmic phase and saturated cultures were harvested and fixed in ethanol overnight at 4°C. Cells were then re-suspended in 50mM sodium citrate, washed and re-suspended again in the same buffer, treated with RNAse A (final concentration= 0.25mg/ml) for 1 hour at 50°C followed with Proteinase K (final concentration= 0.5mg/ml) for 1 hour at 50°C. Cells were then stained with Propidium Iodide solution (final concentration= 16mg/ml) and cell cycle distributions were analyzed using the Epics XL (Beckman-Coulter) flow cytometer. Microscopic measurements of > 30 individual cells were used to calculate cell cycle time (CT) defined as the time at which a cell first budded to the time at which the cell gave rise to the subsequent bud. The overall Cycle Time (CT) was calculated by averaging two mother cell CTs with one daughter cell CT (Table 2). Budding index values were calculated using five μl of the same samples. A minimum of 200 yeast cells were observed microscopically using a phase contrast microscope (Zeiss AxioLab) with a 40X objective. The number of budded and unbudded cells was recorded, and the budding index values were calculated. BLAST software from the National Center for Biotechnology Information (NCBI) was used to identify conserved human homologs.

### Microscopic analysis of cell size

Time-lapse photomicroscopy of > 30 individual cells was used to determine virgin daughter birth size and the “critical cell size” at which the size mutants enter division as described previously [61]. Subsequently, the percent of budded cells was plotted as a function of cell size. Box plots illustrate the distribution of cell sizes for the respective deletion strains. The rectangle includes the range of sizes spanning the first quartile to the third quartile. The horizontal band within the box represents the median value while the whiskers on the top and bottom represent the maximum and minimum values of the range respectively. Mann Whitney statistical tests (GraphPadInStat Version 3.10) were used to evaluate the significance of cell size differences.

### Genetic analyses

Double mutants for epistasis analyses were obtained by mating MAT alpha *cln3* (KanMX::Leu2) and *whi5* (KanMX::Leu2) haploids with MAT a cell size mutants (confirmed by Coulter counter and PCR analyses). At least 2 individual colonies for each double mutant was sized in the logarithmic phase (as described above) and the average calculated as shown in Additional file 1: Table S1. PCR amplification of the unique barcodes was carried out to confirm the respective gene deletions. For over-expression analyses, *GAL* constructs were created using the pYES-DEST52 Gateway Vector System (Invitrogen). Primers for ORF amplification were designed as per the guidelines provided by Invitrogen Life Technologies pENTR Directional TOPO Cloning Kits (Version E). A PCR reaction typically included 100ng DNA template, 2-5μl of Pfu Turbo DNA Polymerase (Stratagene), 100pm of each primer, 8μl of 10X Pfu buffer and 10μl 25mM dNTPs. Standard PCR cycling conditions were: (A) 2 min at 95°C for denaturation, (B) 30 sec at 95°C for denaturation, (C) 30 sec at 50º-55°C for annealing, (D) 1–3 min at 72°C for extension and (E) 5 min at 72°C as the final extension step, with steps (B)-(D) steps repeating 40–45 times. The amplified bands were then excised, from a 1% agarose gel with 0.5μg/ml of ethidium bromide, with the help of QIAEX kits (QIAGEN, Valencia, CA). Cloning procedures for integration into the pYES-DEST52 gateway vector was followed as per Invitrogen instructions. For over-expression of *CTR9*, *GAL1-CTR9* vector was obtained from Thermo Scientific Open Biosystems Yeast ORF Collection with BG1805 as the backbone vector. Due to the large size of *ROM2* (~4Kb), cloning of its ORF was not successful. All *GAL* over-expression vectors were confirmed by restriction digestion. Transformation of yeast was carried out as described previously [86] using -*URA* as a selectable marker. Subsequently, three individual colonies for each were then cultured overnight in -Ura Raf/Gal media (1% Raffinose + 1% Galactose) and the samples were sized in the logarithmic phase. Simultaneously, samples were isolated to calculate the budding index and perform flow cytometry to determine the cell cycle distribution. Rescue experiments were carried out in the respective deletion strains to confirm ORF functionality of over-expression plasmids.

## Competing interests

The authors declare that they do not have any competing interests.

## Authors’ contributions

HD and BLS designed the study. HD drafted the manuscript and carried out the size analysis, over-expression studies, knockout strain construction and epistasis analysis. HH performed the microscopic analysis. JW, LA and JS participated in plasmid isolation, yeast strain constructions and confirmations. TK and AM conducted the over-expression analyses. All authors read and approved the final manuscript.

## Authors’ information

HD is a PhD graduate. JW is a PhD candidate. HH is the laboratory technician. LA is a graduate from Texas Tech University (TTU) with a BS degree in Cell and Molecular Biology. JS is currently pursuing her BS degree in Electrical Engineering at TTU. TK is a pediatric intensivist currently pursuing his MS in Cell and Molecular Biology. AM is a third year medical student at TTUHSC. BLS is an Associate Professor at TTUHSC.

## Supplementary Material

Additional file 1 **Table S1.**The tabs represent the following data. **Hom. LOG**: Average mean, median and mode values for the sizing of 299 homozygous diploid strains in the logarithmic phase. **Het. LOG**: Average mean, median and mode values for the sizing of 468 heterozygous diploid strains in the logarithmic phase. **Hom. SAT**: Average mean, median and mode values for the sizing of 303 homozygous diploid strains in the saturation phase. **Het. SAT**: Average mean, median and mode values for the sizing of 469 heterozygous diploid strains in the saturation phase. **(Hom + Het) LOG**: Average mean, median and mode values for the sizing of 767 diploid strains in the logarithmic phase (299 homozygous diploids + 468 heterozygous diploids). **(Hom + Het) SAT**: Average mean, median and mode values for the sizing of 772 diploid strains in the saturation phase (303 homozygous diploids + 469 heterozygous diploids). **New cell size mutants**: Statistical analysis for cell size of the newly identified size mutants in Table 1. **Size mutant failures**: Statistical analysis for cell size of the 14 mutants which failed to repeat the size phenotypes. **% Repeatability**: Comparative cell size analysis of the overlapping 144 newly constructed deletion strains with the previous screens. **Epistasis size values**: Logarithmic phase mean cell size readings for the epistatic combinations. Click here for file

Additional file 2 **Figure S1.** Cell size analyses of yeast deletion strains. **(A)** Total of 767 deletion strains (homozygous+heterozygous) were sized in the logarithmic phase. Each data point represents the number of strains whose size falls in a 5 fL bin. The two curves represent the geometric mean (diamond) and median (square) of the cell sizes. Upper and lower size limits are indicated by dashed lines and 95% of strains had mean cell sizes within the range of 82.7fL and 118fL (±2SD of the average mean cell size). WT mean cell size (104.1fL) is depicted by the arrow. **(B)** Total of 772 deletion strains (homozygous+heterozygous) were sized in the saturation phase. Each data point represents the number of strains whose size falls in a 5 fL bin. The two curves represent the geometric mean (diamond) and median (square) of the cell sizes. Upper and lower size limits are indicated by dashed lines and 95% had mean cell sizes within the range of 38.8fL and 62.8fL (±2SD of the average mean cell size). WT mean cell size (48.8fL) is depicted by the arrow. Click here for file

Additional file 3 **Figure S2.** Cell size distribution curves in saturation phase. Geometric mean distribution curves are represented for the strains studied. Each data point represents the number of strains whose size falls in a 5 fL bin. **(A)** Screen carried out in the year 2002 (Mean cell size = 50.8 fL). **(B)** Analysis of the new strains (Mean cell size = 50.8fL). Click here for file
