# Supplementary figures and images for: Identification of new cell size control genes in S. cerevisiae
Source: Cell Div. 2012 Dec 12;7:24. doi: 10.1186/1747-1028-7-24 (PMC3541103; doi:10.1186/1747-1028-7-24)

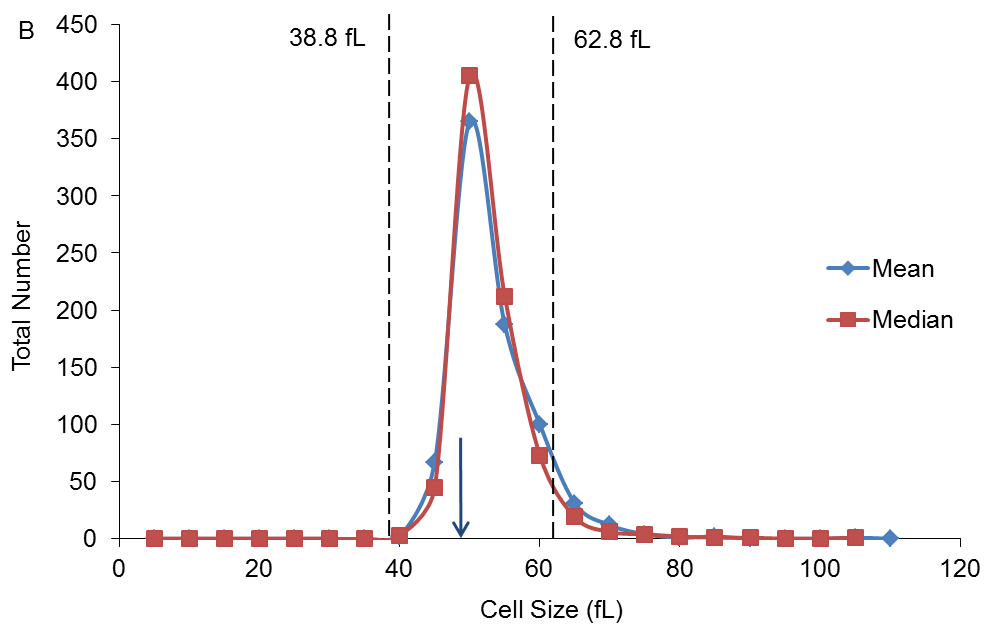

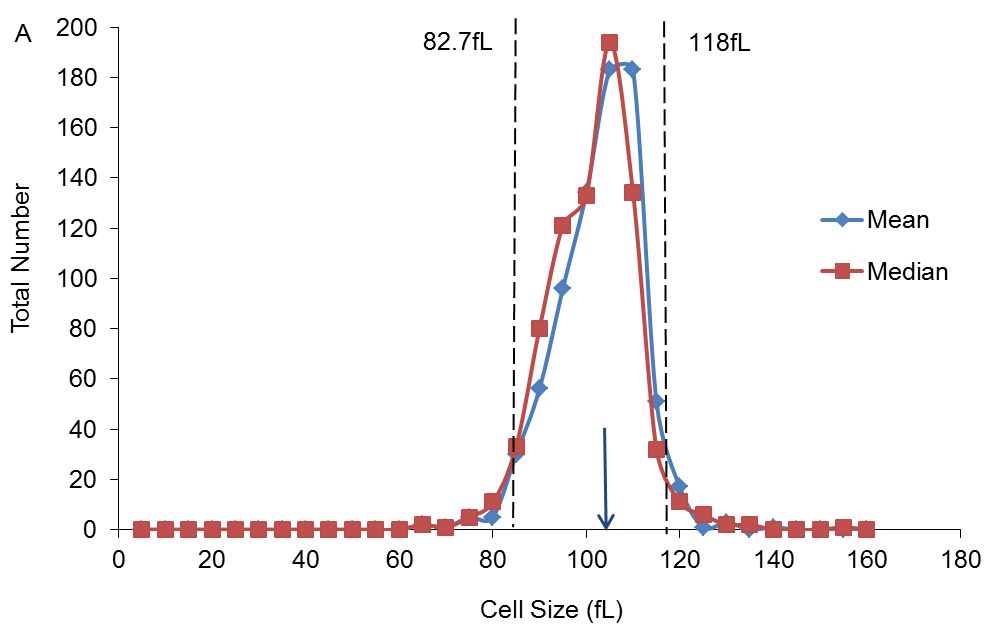

Supplement: Additional file 2 — Figure S1. Cell size analyses of yeast deletion strains. (A) Total of 767 deletion strains (homozygous+heterozygous) were sized in the logarithmic phase. Each data point represents the number of strains whose size falls in a 5 fL bin. The two curves represent the geometric mean (diamond) and median (square) of the cell sizes. Upper and lower size limits are indicated by dashed lines and 95% of strains had mean cell sizes within the range of 82.7fL and 118fL (±2SD of the average mean cell size). WT mean cell size (104.1fL) is depicted by the arrow. (B) Total of 772 deletion strains (homozygous+heterozygous) were sized in the saturation phase. Each data point represents the number of strains whose size falls in a 5 fL bin. The two curves represent the geometric mean (diamond) and median (square) of the cell sizes. Upper and lower size limits are indicated by dashed lines and 95% had mean cell sizes within the range of 38.8fL and 62.8fL (±2SD of the average mean cell size). WT mean cell size (48.8fL) is depicted by the arrow. [file 1747-1028-7-24-S2.doc]

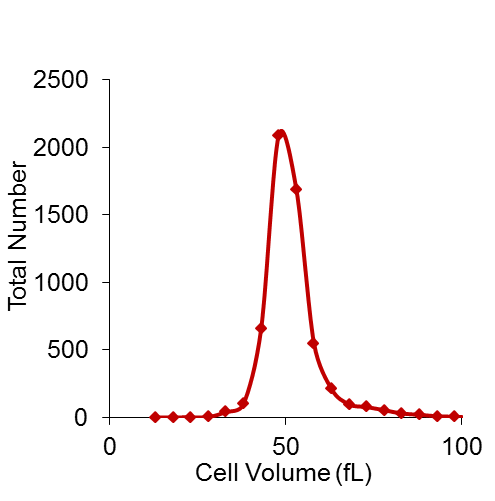

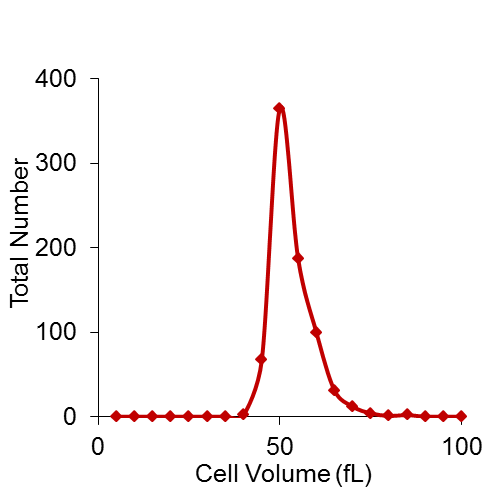


A.

A.

B.

Supplement: Additional file 3 — Figure S2. Cell size distribution curves in saturation phase. Geometric mean distribution curves are represented for the strains studied. Each data point represents the number of strains whose size falls in a 5 fL bin. (A) Screen carried out in the year 2002 (Mean cell size = 50.8 fL). (B) Analysis of the new strains (Mean cell size = 50.8fL). [file 1747-1028-7-24-S3.doc]
